# Insights into the Role of Biopolymer Aerogel Scaffolds in Tissue Engineering and Regenerative Medicine

**DOI:** 10.3390/polym13101612

**Published:** 2021-05-17

**Authors:** Esam Bashir Yahya, A. A. Amirul, Abdul Khalil H.P.S., Niyi Gideon Olaiya, Muhammad Omer Iqbal, Fauziah Jummaat, Atty Sofea A.K., A. S. Adnan

**Affiliations:** 1 School of Industrial Technology, Universiti Sains Malaysia, Penang 11800, Malaysia; essam912013@gmail.com; 2 School of Biological Sciences, Universiti Sains Malaysia, Penang 11800, Malaysia; 3 Department of Industrial and Production Engineering, Federal University of Technology, PMB 704 Akure, Nigeria; ngolaiya@futa.edu.ng; 4 Shandong Provincial Key Laboratory of Glycoscience and Glycoengineering, School of Medicine and Pharmacy, Ocean University of China, Qingdao 266003, China; oiqbal133@gmail.com; 5 Management & Science University Medical Centre, University Drive, Off Persiaran Olahraga, Section 13, Shah Alam 40100, Malaysia; drfauziahjummaat@gmail.com (F.J.); drazreenadnan@gmail.com (A.S.A.); 6 Hospital Seberang Jaya, Jalan Tun Hussein Onn, Seberang Jaya, Permatang Pauh 13700, Malaysia; attysofea.8868@gmail.com

**Keywords:** biopolymers, aerogels, tissue engineering, scaffold, biocompatibility, regenerative medicine

## Abstract

The global transplantation market size was valued at USD 8.4 billion in 2020 and is expected to grow at a compound annual growth rate of 11.5% over the forecast period. The increasing demand for tissue transplantation has inspired researchers to find alternative approaches for making artificial tissues and organs function. The unique physicochemical and biological properties of biopolymers and the attractive structural characteristics of aerogels such as extremely high porosity, ultra low-density, and high surface area make combining these materials of great interest in tissue scaffolding and regenerative medicine applications. Numerous biopolymer aerogel scaffolds have been used to regenerate skin, cartilage, bone, and even heart valves and blood vessels by growing desired cells together with the growth factor in tissue engineering scaffolds. This review focuses on the principle of tissue engineering and regenerative medicine and the role of biopolymer aerogel scaffolds in this field, going through the properties and the desirable characteristics of biopolymers and biopolymer tissue scaffolds in tissue engineering applications. The recent advances of using biopolymer aerogel scaffolds in the regeneration of skin, cartilage, bone, and heart valves are also discussed in the present review. Finally, we highlight the main challenges of biopolymer-based scaffolds and the prospects of using these materials in regenerative medicine.

## 1. Introduction

Injuries, trauma, and even diseases have led to severe tissue damage and degeneration in various human organs; thus, it has always been of great interest to facilitate the repairing process or the regeneration of such damaged tissues. An increasing gap between tissue donation and tissue transplantation has inspired researchers to find alternative approaches for making artificial tissues and organs functional [1]. The global tissue engineering market size in 2019 was computed at approximately USD 9.9 billion and is expected to witness a compound annual growth rate of 14.2% from 2020 to 2027. The potential of tissue engineering procedures in treating irreversible damage of tissues has significantly boosted the market growth [2,3]. Tissue engineering technology provides new hope for therapeutic and cosmetic purposes by growing the cells from the same person, leading to the generation of tissue or even a whole organ, which is highly compatible with the patient’s body. The development of biological substitutes allowed for the potential regrowth of organs, which is still an interdisciplinary scientific field of research [4]. Regenerative medicine has been defined by the United States National Institutes of Health as the method of creating living, functional tissues or organs to repair or replace damaged tissue or organ due to age, disease, accident, damage, or even congenital defects [5]. Aerogel is a low-density nanoporous solid material with an ultrafine open pore structure, resulting in extremely large surface areas and low densities [6]. Aerogels have been prepared from various materials, including biopolymers [7,8], resulting in a desirable combination of properties suitable for many non-medical applications such as water purification [9,10,11,12], thermal insulation [13], acoustic properties [14], etc., and medical applications such as drug delivery [15,16], biosensing [17,18], tissue scaffolding, and regenerative medicine [19,20]. Various technological approaches have been developed to fabricate aerogel scaffolds, including freeze-drying, phase separation, particulate-leaching, and gas foaming, showing inexpensive approaches and optimized physicochemical property structures [21]. Regenerative medicine has significantly evolved in recent years with the 3D bio-printing of various biopolymeric combinations, which has shown promising potential in restoring the function of diseased tissues and organs [22].

The function of all human cells occurs at a nanometer scale. Thus, nanobiotechnology has mimicked the natural environment surrounding the cells to enhance their growth, proliferate, and interact, and has led to the regeneration of tissues or even a whole organ within the porous aerogel scaffold [23]. Biopolymer-based aerogels have been widely investigated and reviewed for their properties [24], fabrication techniques [25], biocompatibility [26], and cytotoxicity [27,28]; many studies have revealed their potential in tissue engineering as well as other biomedical applications [25,29,30,31]. Previous reviews either explain a single type of tissue regeneration such as skin [32], bone [33], cartilage [34], or valves [35], and many of these reviews focus on a particular scaffold-based material such as chitosan [36] or cellulose [37]. Furthermore, biopolymer hydrogel-based scaffolds have been extensively reviewed in many previous reviews, including [38,39]. Here we review recent advances in the role of biopolymer aerogel scaffolds in tissue engineering and regenerative medicine, going through the principle of tissue regeneration and the desirable characteristics in biopolymers that have attracted researchers to use them in tissue regeneration instead of other materials. This review also presents the latest work in terms of regenerating skin, cartilage, bone, and heart valves using biopolymeric-based scaffolds and highlights the main challenges and future prospects of these materials in tissue engineering and regenerative medicine.

## 2. Tissue Scaffolding and Regenerative Medicine

The past few years have witnessed great advances in the fabrication of different scaffolds able to provide a proper micro-environment for the proliferation and interaction of various human cell types, leading to the formation of tissue or whole organs [40]. A tissue engineering scaffold is a material that has been engineered to enhance cellular growth and cause desirable interactions between the cells, leading to the formation of functional tissue for medical purposes [41]. The growing cells are often “seeded” into the scaffolds, which should be designed to support the formation of three-dimensional tissue structure and not cause any cytotoxicity or genotoxicity [4,42]. The ideal scaffold should allow for the transportation of the nutrients to the cells, which is necessary for their attachment, proliferation, and differentiation, and provide mechanical support to the cells and control the degradation rate without showing any cytotoxicity or signs of inflammation [43].

### 2.1. Chronological Development of Tissue Scaffolding and Regenerative Medicine

Tissue engineering is a relatively recent field that uses biocompatible scaffolds, living cells, and suitable biochemical and physical factors such as growth factors and cyclic mechanical loading to create tissue-like structures suitable for transplantation. It all started as early as 600 BC when Indians treated mutilations of the nose, ear, and lip by using free gluteal fat and skin grafts [44]. They used subcutaneous fat taken from the gluteal region and secret cement for fixation and adhesion. In 1442, an Italian surgeon, Brancas, successfully transplanted a nose taken from a slave to his master using a graft from the arm. Hundreds of years later, Bunger of Marburg reported in 1823 a partly successful transplantation approach of a free whole-thickness thigh skin graft for the repairing of nasal defects [45]. Table 1 presents an illustration of the chronological evolution of tissue engineering and regenerative medicine.

**Figure 1:**
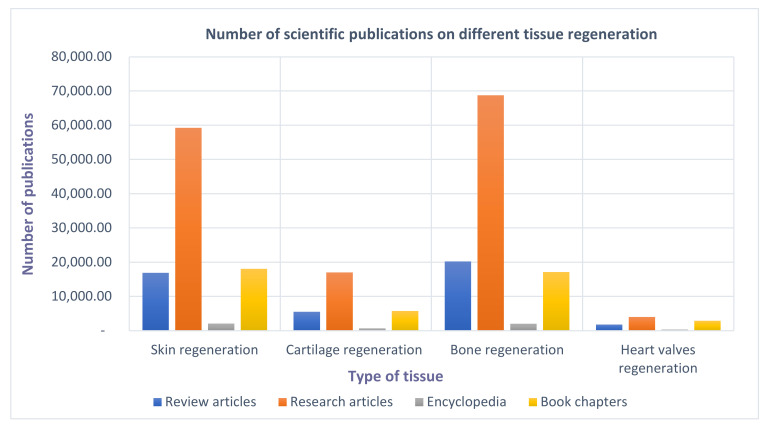
Number of scientific publications on different tissue regeneration (search done through Science Direct on 1 May 2021).

The first attempt of the fabrication of synthetic skin substitute was developed in 1962; however, the first successful trial was achieved in the late 1970s and early 1980s, which is considered as the modern era of tissue engineering and regenerative medicine, although the term “tissue engineering” was coined around 1987 [67]. Since then, a significant amount of research has been conducted to regenerate different tissues, as indicated by the number of publications on the regeneration each of skin, cartilage, bone, and heart-valves (Figure 1). Skin and bone regeneration gained the most interest of researchers due to their simplicity compared to heart valves, which required specialized structures [35]. However, with the development in material science and fabrication techniques, particularly the use of 3D printing technologies to print biopolymer-based scaffolds, many specialized and highly accurate scaffolds have been successfully fabricated for the regeneration of most body tissues, including heart-valves [68].

### 2.2. Tissue Regeneration Approaches

**Figure 2:**
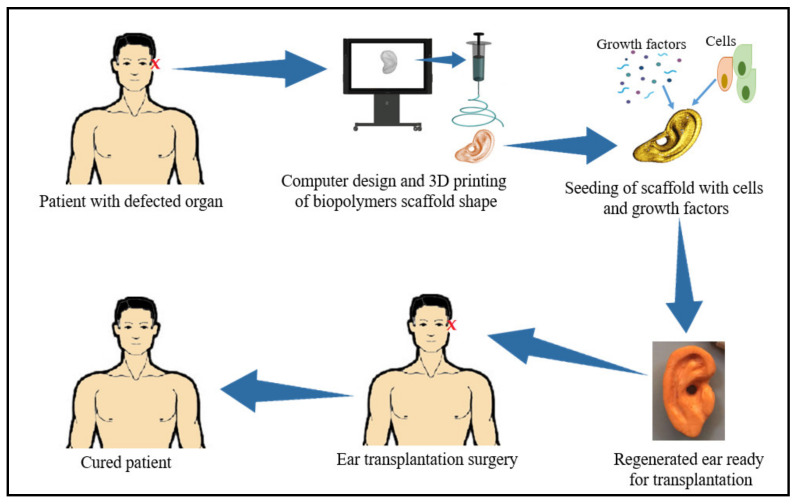
Schematic illustration of the tissue engineering process.

Two main approaches have been used in tissue engineering technology, namely acellular and cellular approaches; acellular approaches involve the use of natural or synthetic matrices to help new tissue growth and encourage self-repairing of the body’s tissue using its natural ability [69]. The cellular approaches involve using donor cells or tissue either alone or implanted into a biocompatible scaffold for new tissue formation [70]. Stem cells are the main and major supplying source of human cells for most regeneration applications, mostly supplied with growth factors that promote their transformation to different types of cells [71]. The goal of tissue engineering is to restore, maintain, or fix the biological function of the particular damaged tissues or whole organs. This process involves reviving cells and/or tissues from their natural biological environment, followed by their in vitro growth and proliferation using suitable scaffold growth factors for the desired tissue [72,73]. Finally, the ready tissue or organ are reintroduced into the biological micro-environments, as summarized in Figure 2. The concept of tissue engineering has been used beyond therapeutic and cosmetic purposes, including biosensing, monitoring, and diagnostic intentions [72,74].

## 3. Biopolymer-Based Aerogels in Tissue Engineering and Regenerative Medicine

Naturally occurring polymers (biopolymers) are obtained from various renewable resources, including plants, animals, and microorganisms, which are similar to the natural biological macromolecules and much easier to be recognized by the environment than synthetic ones [75]. Different biopolymers have been clinically used for tissue engineering and implant fabrication, including polysaccharides (e.g., chitosan, alginate, chitin, gellan gum, and derivatives), and proteins (e.g., collagen, silk fibroin, gelatin, keratin, actin, and elastin), in addition to some glycosaminoglycans such as hyaluronic acid [21,76]. Due to their high similarity with the extracellular matrix, biopolymers may elude chronic inflammation toxicity or even immunological reactions, which are frequently noticed with synthetic polymers [25]. The unique characteristics of biopolymers, such as their high biocompatibility, non-cytotoxicity, non-genotoxicity, molecular weight, degree of branching, and composition, have made them attract the attention of researchers in tissue engineering applications [77]. An aerogel can be defined as a solid, loose, ultra-lightweight, and lucid open porous network, which can be obtained from a variety of precursors, including biopolymers, by removing the pore liquid without affecting the network structure [78]. The type and concentration of these biopolymers have significant effects on both the porous network of the aerogel on a molecular level and the bulk properties and thus the viability and proliferation of growing cells [79,80].

### 3.1. Desirable Characteristics in Biopolymers and Biopolymers Tissue Scaffolds

Biopolymers with abundant functional groups are regarded as important and attractive ingredients for tissue scaffold fabrication [81]. The high biocompatibility, non-genotoxicity, and non-cytotoxicity of biopolymers either in solo state or composites, coupled with the variety of chemical and biological functionalities they possess, result in them being promising scaffolds for tissue engineering technology [82,83,84]. Biocompatibility is referred to as the ability of the material to interact and function in living tissue/s without any complications [85]. The biocompatibility of any material mostly depends on its cytotoxic induction to certain or all cell types in addition to the immunological response upon its exposure to our body fluids or cells. Table 2 presents a summary of the literature investigating the biocompatibility and cytotoxicity of some biopolymer tissue engineering scaffolds. However, combining these biomaterials with other inorganic materials or synthetic polymers may reduce their biocompatibility and thus affect the growing of cells [80,86].

Nature offers a variety of biopolymers with multiple attractive functions and beneficial properties for tissue engineering; bioresorbability, as an example, is the ability of a material to degrade within the host tissue at the appropriate rate and is a highly desirable property that can only be found in some biopolymers but not in industrial or inorganic ones [97,98,99]. The regeneration of rough tissues such as cartilage and bone requires tunable Poisson’s ratio scaffolds. These tissues can imitate the growth environment and significantly affect the proliferation of chondrocytes and osteocytes under external environmental stimulation [100]. Song et al. [101] found that tissue scaffolds with negative Poisson’s ratios could significantly promote vascular differentiation marker expression in addition to the secretion of the extracellular matrix protein vitronectin. The porosity of tissue scaffolds is an important characteristic for growing cells and their later interactions. In a recent study, Tang et al. [102] used a mix of cellulose nanofibers and polyethylene glycol diacrylate to fabricate aerogel for tissue scaffolding. The authors used polyethylene glycol diacrylate as a photocurable resin to crosslink with the cellulose nanofibers and form hydrogels with customized pore structures; however, cellulose nanofiber significantly contributed to the porosity of the resulting aerogels after freeze-drying. The three-dimension of the tissue scaffold should be adequately designed in terms of architecture as well as physicochemical properties. Some biopolymeric aerogels can dissolve in aqueous solution and transform into soft hydrogels and have been found to be good candidates to mimic some tissue environments [103,104]. Mahumane et al. [105] suggested that the tissue scaffold should be designed with pores that are small enough to support three dimensions of cell–cell contacts and at the same time large enough to allow nutrients, oxygen, and bioactive factor diffusion to ensure the survival and growth of cells. Many studies reported the ability to control the porosity and the pore size of biopolymer-based aerogels; pore sizes between 20 and 160 μm were adequate for cell growth, proliferation, and colonization [105,106]. The unique biological properties and the ability to customize the scaffold architecture have made the biopolymeric aerogels of great interest among researchers in the past few years to develop novel solutions for many tissue defects.

### 3.2. Fabrication Techniques of Biopolymeric Scaffold for Tissue Engineering

**Figure 3:**
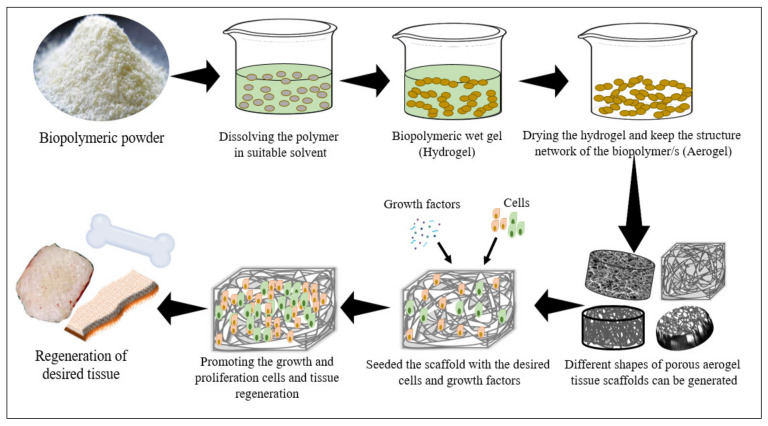
Schematic illustration of the original principle of using biopolymer aerogel scaffolds in tissue regeneration.

Since the discovery of aerogels back in 1931 by Samuel Stephens Kistler, they have been utilized for different applications, including tissue scaffolding [107]. The 3D biopolymer aerogel scaffolds arose because highly porous matrices can provide a proper micro-environment for cells to grow and increase normally. The ability to adjust the pore size and the porosity of these biopolymeric scaffolds permit their customization to suit different tissues [108]. Tissue scaffolds should permit the transport of needed oxygen, nutrients, and growth factors for the growth, attachment, proliferation, and differentiation of cells. Some biopolymeric scaffolds have been reported to enhance the attachment of cells to the scaffold and stimulate cell–biomaterial attachment, growth, and migration [106,109]. A variety of methods has been used in aerogel tissue scaffold fabrication. Most of the initial techniques, such as freeze-drying, gas foaming, solvent casting, and practical leaching, etc., follow the original principles of the sol–gel approach [76]. Figure 3 presents a schematic illustration of the sol–gel approach for the fabrication of biopolymer-based aerogels for the regeneration of different tissues.

Lately, the use of 3D printing and rapid prototyping machines such as electrospinning, stereolithography, and selective laser sintering has allowed for the fabrication of highly accurate shapes of biopolymeric aerogel scaffolds to suit the desired tissue to be regenerated [110]. Electrospinning is one of the most used techniques for biopolymeric scaffold fabrication, producing the desired shapes through coaxial electrospinning [111]. Movahedi et al. [112] used a novel electrospinning technique to fabricate biopolymer nanofiber-based scaffolds for skin tissue engineering and reported many advantages of these fabrication techniques. Freeze-drying is used in most cases, even after advanced techniques to remove the solvent of undried scaffolds [113]. Table 3 summarizes the most used techniques for biopolymeric aerogel scaffold fabrication for tissue engineering applications.

## 4. Biopolymers-Based Aerogels in Tissue Engineering for Therapeutic Applications

With the increasing need for more advanced therapeutics for tissue engineering and regenerative medicine, 3D biopolymer aerogel scaffolds have arisen as highly porous matrices that are easy to customize to different shapes and that provide a proper micro-environment for the proliferation and interaction of cells, leading to the formation of tissue or organ. Cellulose nanocrystal-based 3D aerogels possessing excellent biocompatibility and mechanical properties have been produced using direct ink writing technology followed by freeze-drying [122]. The aerogel can be customized to different shapes to suit the desired part of the body without structural collapse or shrinkage and then seeded with the desired cells and growth factors to promote the differentiation of the seeded cells and generation of desired tissues.

### 4.1. Wound Healing and Skin Regeneration

**Figure 4:**
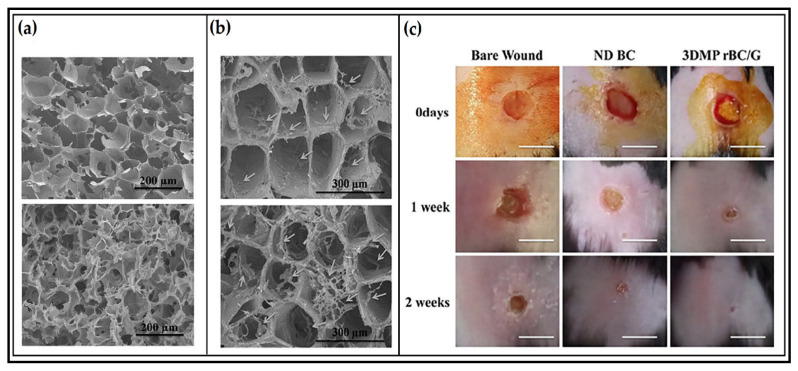
Illustration of the 3D micro-porous regenerated bacterial cellulose/gelatin (3DMPrBC/G) tissue scaffold: (**a**) SEM images of the scaffold, (**b**) cell adhesion and proliferation after 3 days and 7 days of incubation, (**c**) experimental in vivo skin regeneration. Adapted with permission from ref. [125]. 2018 Elsevier.

Treatments of skin injuries such as cuts, lacerations, tears, scratches, and burns caused by trauma or diseases are among the most critical problems that could lead to further issues. Recently, different types of biopolymer-based scaffolds and aerogels have been used for tissue engineering to cover the wound area, which acts as dressing material and artificial skin simultaneously by mimicking the physicochemical properties of the extracellular matrix of the patient’s native skin [123]. Abdul Khalil et al. [124] prepared gelatin, cellulose acetate, and elastin-based scaffolds using the electrospinning technique for skin regeneration and wound healing applications. The authors reported that in vitro experiments showed that the prepared biopolymeric scaffolds significantly supported the growth, attachment, and proliferation of human fibroblast cells. Similarly, Khan et al. [125] fabricated a 3D micro-porous regenerated bacterial cellulose/gelatin tissue scaffold for the regeneration of skin tissues. The prepared aerogel showed excellent adhesion and proliferation potential of human keratinocytes on the surface and within the structures of the scaffolds during one week of incubation. The authors characterized their scaffold using confocal microscopy and observed penetration of human keratinocytes into the scaffolds. Furthermore, the wound healing and skin regeneration experiments were done using experimental mice, which showed complete skin regeneration with wound closure efficacy of 93%, which was much higher than that of pure BC-treated (63%) wounds or the control (47%). Figure 4 presents the structure of the prepared scaffold, and the excellent adhesion and proliferation potential of human keratinocytes and the wound healing experiments.

**Figure 5:**
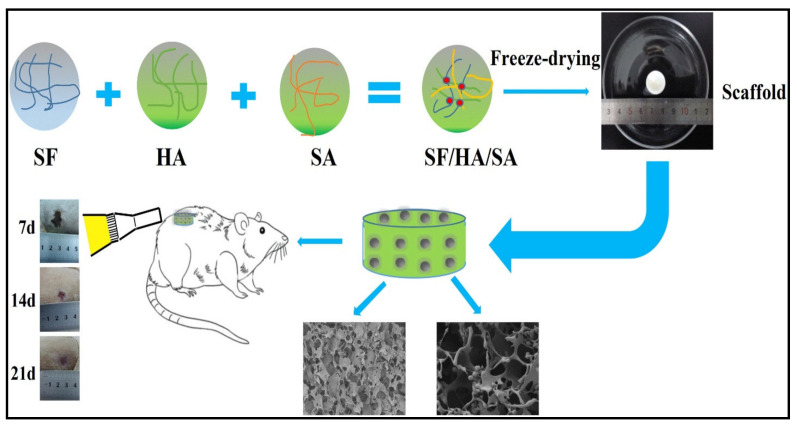
Schematic illustration of silk fibroin (SF), hyaluronic acid (HA), and sodium alginate (SA) composite scaffold for wound dressing and skin regeneration applied on an animal full-thickness burn model. Adapted with permission from ref. [130]. 2019 Elsevier.

Many studies have reported that the structure of biopolymer nanofibers resembles the dermal extracellular matrix. Thus, the presence of these nanofibers with high-interconnected porosity significantly promoted efficient cellular infiltration, growth, and proliferation from the initial days of cell seeding [32,126]. Badami et al. [127] showed that human osteoprogenitor cells could adhere, grow, and increase on random fused fiber topographies, with a mean range of fiber diameters of 0.14 nm to 2.1 μm. Ghaee et al. [128] fabricated a biomimetic structure for wound healing and skin regeneration using biopolymer composite hydrogels loaded with curcumin. The authors reported proper biocompatibility and good attachment of cells to the prepared scaffold, confirming the suitability of biopolymers for tissue regeneration. Sun et al. [129] used a different strategy for wound healing by incorporated two essential extracellular matrix proteins, fibrinogen and collagen I, into the shell and the core of a nanofiber scaffold, respectively. The authors used these proteins to mimic their sequential appearance in the wound healing process. The biomimetic coaxial scaffolds were found to remarkably promote the immune-modulatory paracrine secretion of adipose-derived mesenchymal stromal cells. The same authors incorporated macrophages with adipose-derived mesenchymal stromal cell-conditioned medium. They observed enhanced immune modulation of the stem cells on the biomimetic coaxial polymeric scaffold, which was confirmed by the enhanced polarization of the macrophages, leading to effective promotion of wound repair by resolving the inflammation of the wound. Yang et al. [130] aimed to mimic the natural structure of the skin’s extracellular matrix by using a composite aerogel scaffold consisting of silk fibroin, hyaluronic acid, and sodium alginate, which exhibited high porosity and elastic characteristics. The authors reported that the composite aerogel scaffold showed better attachment, growth, and proliferation of fibroblast cells than binary biopolymeric blends. The wound healing effects of the scaffolds were done in a rat full-thickness burn model (Figure 5), which showed significant improvement of re-epithelialization and enhanced extracellular matrix remodeling after the application of the scaffold on the wound [130]. The biocompatibility of natural polymers and the role of hyaluronic acid to retain water keep the cells and formed tissues well lubricated, providing the perfect situation for the cells to increase, which promotes wound healing and accelerates skin regeneration.

### 4.2. Cartilage Regeneration

**Figure 6:**
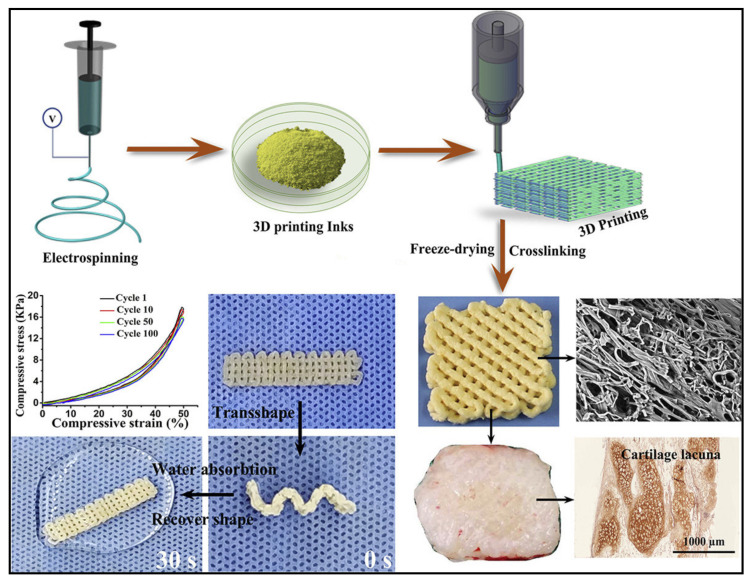
Schematic illustration of 3D printed electrospun scaffold for cartilage regeneration. Adapted with permission from ref. [137]. 2019 Elsevier.

Articular cartilage repair is still considered a huge challenge for scientists and clinicians. However, the great advance in material science and 3D bioprinting technologies has allowed for the fabrication of biopolymer scaffolds for cartilage tissue engineering. Yang et al. [131] used type I collagen mixed with sodium alginate as bio-inks and then incorporated chondrocytes (cartilage cells) to construct 3D bioprinted cartilage tissue. The authors reported that their biopolymer scaffold distinctly facilitated the adhesion of chondrocytes, accelerated the proliferation, and enhanced cartilage-specific gene expression, indicating that the printed scaffold effectively preserved the phenotype and suppressed dedifferentiation of chondrocytes. It has been proven that scaffolds with pore sizes between 250 and 500 µm lead to better cartilage repair, resulting from enhanced proliferation, differentiation, and extracellular matrix production ability [132]. Shi et al. [133] designed a silk fibroin–gelatin scaffold with a uniform 350 µm pore size and a three-layer height using 3D printing technology to match the exact thickness of rabbit articular cartilage. The authors reported that their optimized scaffold showed superior cartilage repair performance in rabbit knee joint due to their not retaining adequate for bone marrow stem cells as the efficient recruiting ability of the optimized scaffold, which acted as a physical barrier for blood-clotting and provided the mechanical protection before neocartilage formation and a suitable 3D micro-environment for bone marrow stem cell proliferation, differentiation, and extracellular matrix production [133]. Nguyen et al. [134] investigated the potential use of nanofibrillated cellulose with alginate or hyaluronic acid as a bio link for cartilage regeneration. The authors reported that using nanofibrillated cellulose with hyaluronic acid showed markedly low proliferation and phenotypic changes in the cells away from pluripotency. However, using nanofibrillated cellulose with alginate constructs, pluripotency was initially maintained, and a marked increase in cell number was also observed by 2-photon fluorescence microscopy. The authors also reported that hyaline-like cartilaginous tissue was observed after five weeks with the expression of collagen type II and lacking tumorigenic expression of Oct4. A tunable Poisson’s ratio aerogel scaffold composed of cellulose nanofibers and polyethylene glycol diacrylate was used to simulate the mechanical behavior of natural tissues and cartilage regeneration. The negative Poisson’s ratio scaffold impressively provided a good environment for the growth and proliferation of bone marrow mesenchymal stem cell (mBMSC) and chondrogenic induction [102]. Electrospun nanofibers have been widely used in the past few years for various biomedical applications, including tissue engineering and regenerative medicine [135,136]. Chen et al. [137] combined three-dimensional printing and freeze-drying to fabricate 3D scaffolds with large pores with precisely controlled shapes using pure gelatin fibers and poly (lactic-co-glycolic acid). The fabricated scaffolds possessed good elasticity, water-induced shape memory, and fibrous surface morphologies similar to those of a native extracellular matrix. The authors found that the scaffold was immediately combined with chondrocytes and attained satisfactory in vivo cartilage regeneration. Figure 6 presents a summary of the steps of scaffold fabrication and the final results of the cartilage.

The preparation of electrospun biopolymeric fibers into three-dimensional scaffolds with similar characteristics to the native extracellular matrix in terms of pores size, surface chemistry, and the 3D shape is essential for tissue regeneration. Lahann et al. [138] developed a novel fabrication process referred to as 3D jet writing, an advanced electrospinning technique that can precisely control the pore size and geometries of the scaffolds. Chen et al. [139] fabricated 3D gelatin/PLA nanofiber-based aerogels for cartilage tissue scaffolding using the electrospinning technique. The authors further crosslinked their scaffold with hyaluronic acid to improve the repairing effect of cartilage, and they cultured chondrocytes on the modified 3D scaffold. The results indicated excellent cytocompatibility and superabsorbent properties of all the scaffolds with elastic characteristics in the wet state. The addition of hyaluronic acid significantly enhanced the repair of cartilage, suggesting great potential for biopolymer aerogels in cartilage regeneration [139].

### 4.3. Bone Regeneration

**Figure 7:**
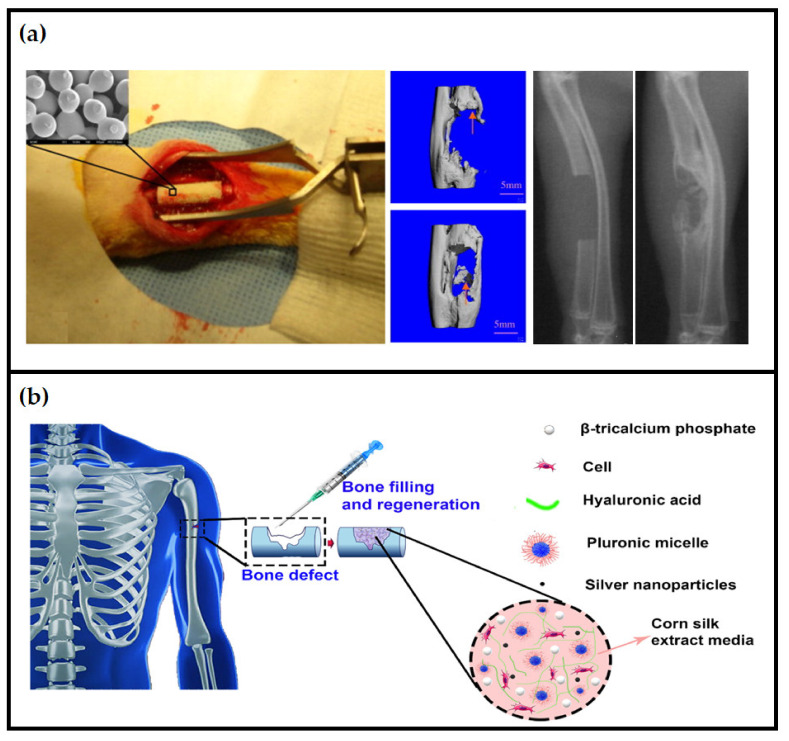
Biopolymer-based scaffolds in bone regeneration: (**a**) implantation of a chitosan/poly(lactide-co-glycolide) scaffold in a 15 mm surgical induced ulna defect and bone regeneration after 12 weeks showing the formation of bridges (adapted Adapted with permission from ref. [144]) 2010 Elsevier; (**b**) injecting the thermo-sensitive hyaluronic acid/corn silk extract composite scaffold containing β-tricalcium phosphate in defected bone for its regeneration (adapted with permission from ref. [95]) 2020 Elsevier.

Despite the excellent ability of bone tissue for self-repair of small defects, their healing capacity has some defects due to trauma or some particular diseases that may lead to the non-union of bone [140]. Therefore, the use of grafts and bone substitutes is necessitated in these situations to aid in healing. Tissue scaffolds play a critical role in bone regeneration by providing structural maintenance of the exact defected region, carrying therapeutically-relevant factors and contributing a void space for vascularization and tissue infiltration [141]. Biopolymer-based scaffolds have been used in bone regeneration due to their high compatibility and degradability, two essential properties in bone tissue engineering scaffold [25]. The scaffold of bone regeneration should be completely resorbed and degraded by the time the defect is regenerated. Tunable bioresorbability, non-cytotoxicity, and protein adhesion of many biopolymers make them favorably used in bone regeneration. In recent work, Huang et al. [142] showed that hydroxyapatite/crystalline nanocellulose-based scaffolds have potential as bone tissue scaffolds through in vitro preliminary protein adhesion investigations and simulated body fluid testing. Osorio et al. [108] evaluated cellulose nanocrystal aerogels with osteoblast-like Saos-2 cells for bone regeneration applications and found a significant increase in the cell’s metabolism. Submerging the aerogels in simulated body fluid solution resulted in the demonstration of hydroxyapatite growth over 14 days. Sulfated cellulose nanocrystal aerogels showed a significant increase in bone volume fraction and evidence of osteoconductivity, concluding the ability of the aerogel in facilitating bone growth and cell proliferation after they are implanted in bone defects. Wei et al. [143] used bioink consisting of silk fibroin, hyaluronic acid, gelatin, and tricalcium phosphate to print 3D silk fibroin-based hybrid scaffolds and treated them with human platelet-rich plasma. The authors reported that the hybrid scaffold promoted the growth and proliferation of human adipose-derived mesenchymal stem cells and significantly up-regulated late osteogenic marker gene expression. In the research of Jiang et al. [144], the authors investigated the degradation properties of the chitosan/poly(lactide-co-glycolide) scaffold and its potential in bone regeneration capacity in a rabbit model having ulnar critical-sized defects (Figure 7a). The prepared scaffolds were able to promote bone formation and regeneration in the rabbit ulnar critical-sized defect model. Successful bridging of the critical-sized defect was observed through micro-computed tomography analysis on the sides both away from the radius and adjacent to it, which occurred using chitosan/poly(lactide-co-glycolide)-based scaffolds.

Furthermore, the histological analysis of the regenerated bone suggested that the scaffold promoted and supported normal bone formation through the intramembranous formation. Makvandi et al. [95] used injectable hydrogels composed of hyaluronic acid and corn silk extract–nanosilver and containing β-tricalcium phosphate for bone regeneration (Figure 7b). The authors seeded their prepared scaffold with mesenchymal stem cells and reported high bone differentiation, suggesting great potential for biopolymer as a potential scaffold for bone tissue regeneration.

### 4.4. Heart Valve Regeneration

Tissue-engineered scaffolds have offered the potential of regeneration of various tissues, including more complex ones such as heart valves. The engineering of heart valve tissues is an alternative approach for the conventional permanent regenerative valve replacement [145]. The scaffold is designed to promote tissue regeneration. The endogenous mechanisms that drive the formation of tissue and remodeling, and the slow degradation of the biopolymeric scaffold make no need for any removal operation [146]. Wang et al. [147] designed collagen and elastin 3D hydrogel scaffolds to mimic the native extracellular matrix of heart valves. The authors encapsulated interstitial valve cells into the hydrogels and valve endothelial cells, which were cultured onto the surface of the hydrogel to create an in vitro three-dimension valve endothelial cell–valve interstitial cell co-culture. Over seven days, the expression levels of F-actin and integrin β1 in valve interstitial cells stabilized, and the cells continuously increased, with significant elongation in their morphology. However, valve endothelial cells initially mediated low expression of integrin β1 and F-actin, and 20% of the cells transformed to the mesenchymal phenotype after the 7th day with higher expression of integrin β1 and increased actin filaments [147]. In a different study, Fu et al. [148] fabricated a degradable chitosan–collagen composite scaffold and seeded it with cells for heart valve regeneration. The seeded cells were smooth muscle cells, endothelial cells, and fibroblasts, which were confirmed by staining techniques and histological analysis. The authors reported that the content of 6-ketone prostaglandin, as measured by radio-immunoassay, of the collagen–chitosan cell culture fluid was significantly higher than that of the serum-free medium, suggesting great potential for collagen–chitosan composite scaffolds for supporting the growth of heart tissues.

**Figure 8:**
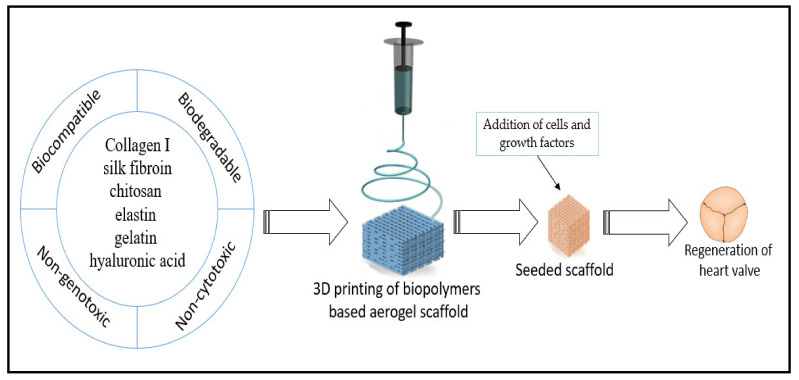
Schematic illustration of heart valve regeneration using biopolymer aerogel-based scaffolds.

Jahnavi et al. [149] used decellularized bovine pericardium–polycaprolactone–chitosan to fabricate an aligned nanofibrous bio-hybrid scaffold for heart valve regeneration. Different human cells were seeded on the prepared bio-hybrid scaffolds, and dense extracellular matrix deposition was observed after a few days, indicating the growth and proliferation of all seeded cells on the scaffolds. The authors conducted uniaxial mechanical tests for the regenerated valve along the axial direction. They reported that the scaffolds were at least 20 times stronger than the native valves and had nearly three times more stiffness than native valves. Similar results were obtained in the study of Du et al. [150], who used a 3D structure scaffold composed of silk fibroin and nanofibrous poly(ester urethane) urea for heart valve tissue engineering. The authors reported that their composite scaffolds significantly supported seeded human umbilical vein endothelial cell growth, suggesting promising potential of the biopolymeric scaffolds for future development and the regeneration of heart valves. Figure 8 presents a summary of the steps of heart valve regeneration using biopolymer aerogel-based scaffolds.

## 5. Challenges and Future Prospects in Tissue Engineering Applications

The preparation process of tissue engineering scaffolds has been steadily developed over the years. Many recent attempts have successfully prepared scaffolds using natural precursors to avoid any possible short-term or long-term toxicity. The construction of the scaffold–tissue complex is a relatively complex process and sometimes requires new optimization when using different cells or different materials. Thus, some researchers have tried to store spare sensitive tissues such as heart valves by a de-cellularizing process, which can be re-cellularized before or after implantation [151,152]. Growth factors or bioactive factors are commonly-used substances in almost all tissue engineering applications [153]. However, some of these factors have been reported with poor stability and a very short half-life [154], which may cause the spreading of these materials to other body parts upon the transplantation. Not much has been done yet on the possible interaction between different growth factors and the biopolymeric scaffolds, which is still considered a major challenge. Many biopolymers and two-dimensional nanomaterials have shown great potential to increase their mechanical properties for future rough tissue engineering applications such as bone, cartilage, ligaments, and tendons. Upon using tissue scaffolds for skin and wound regeneration, the risk of scar tissue was demonstrated in some biopolymers such as silk fibroin [155]; thus, more research needs to be done to move towards safe clinical trials and approved products without any side effects based on these excellent biomaterials. Regeneration of rough tissues has other challenges, such as the kind of mineral content in bone tissue scaffold, which has an optimum in osteogenesis and an effect of the content on bone repair [156]. In most regeneration processes, parameter optimization needs to be further investigated to have a more in-depth understanding of these obstacles. Thus, we may conduct further research on how to balance between cells, growth factors, and scaffolds. The future of tissue engineering technology will witness the use of 4D printing technology of biopolymer-based scaffolds, with the normal 3D printing combined with time, to overcome some of the limitations associated with 3D printing technology, such as the optimization of cell–construct interaction functional responses [157] and the sophisticated dynamics of native tissue fabrication [158]. Therefore, biopolymers could be key biomaterials as bioink formulations, illustrating their tremendous potential in future 4D bioprinting for tissue engineering and regenerative medicine.

## 6. Conclusions

Scaffolds with gradients of physicochemical properties and controlled 3D architectures are crucial for tissue engineering. These specialized structures can be produced using biopolymer aerogel scaffolding. The rapid prototyping and 3D printing technique allows for the fabrication of desired structures of biocompatible aerogel scaffolds from different biopolymers. The unique properties of the biopolymers and the structured features of aerogels have led to great advances in the regeneration of various human tissues. Biopolymer aerogel scaffolds have distinct advantages, and in vitro and in vivo testing have produced positive results for cell attachment, proliferation, and angiogenesis. This review presented the principle of tissue regeneration and the role of biopolymer aerogel scaffolds in the regeneration of skin, bone, cartilage, and heart valves and the main challenges associated with tissue engineering and regenerative medicine. Biopolymer aerogel scaffolds should be the future biomaterial and the main direction of tissue engineering scaffold development using nanoscale fabrication techniques. These innovative scaffolds can solve the major obstacles associated with synthetic polymers and inorganic scaffolds, including cytotoxicity and biocompatibility issues. Biopolymer aerogel scaffolds have promising potential to be the main precursor for current and future tissue engineering scaffolds.

## Figures and Tables

**Table 1:** The chronological evolution of tissue engineering and regenerative medicine.

Scientist/s and Year	Type of Tissue	Scaffold Material	Remark	Ref
Indians in 600 BC.	Skin and cartilage.	Free gluteal fat.	Using secret cement for adhesion.	[45]
Brancas in 1442.	Nose cartilage.	Isograft.	The nose of slave to his master.	[46]
Boronio in 1804.	Skin substitute.	Autograft.	Auto-graft of full-thickness skin grafts on a sheep.	[47]
Bunger in 1823.	Skin tissues.	Autograft.	Skin is taken from the thigh for the repair of nasal defects.	[45]
Alexis Carrel in 1911.	Endothermal animal cells.	Thin layer of clotted plasma.	Recipient of Nobel Prize in Medicine for tissue culture.	[48]
Blakemore et al. in 1954.	Vascular graft.	Silk handkerchief and Vinyon.	The first prosthetic vascular graft implanted in a human patient.	[49]
Per Ingvar Brånemark in 1960s.	Bone tissue.	Titanium cylinder.	The establishment of the osseointegration concept.	[50]
W. T. Green in the 1970s.	Cartilage tissue.	Spicules of bone.	Seeding cells onto spicules of bone and implanting them in nude mice.	[51]
Vacanti et al. in 1988.	Different fetal and adult rat and mouse cells.	Polyanhydrides, polyglactin 910, and polyorthoester.	Successful transplantation of cells in synthetic biodegradable polymers.	[52]
Stone et al. in 1997.	Meniscal cartilage.	Collagen-based scaffold.	No adverse immunological reactions were reported.	[53]
Zein et al. in 2002.	Different human tissues.	Bioresorbable polymer.	Fused deposition modelling used for aerogel scaffold fabrication.	[54]
Svensson et al. in 2005.	Cartilage tissue.	Bacterial cellulose scaffold.	Concluded a high potential for this biopolymer in tissue regeneration.	[55]
Macchiarini et al. in 2008.	Engineered trachea.	Decellularized matrix of human donor trachea.	Removing all the antigens from donor trachea and seeding it with human stem cells.	[56]
Norotte et al. in 2009.	Various vascular cell types.	Direct bioprinting.	Fully biological self-assembly approach for tissue engineering.	[57]
Ahn et al. in 2010.	Skin tissue regeneration.	3D collagen scaffolds.	The scaffold supported the migration and infiltration of cells.	[58]
Zhou et al. in 2013.	Bone tissue.	Bio-nanocomposite scaffolds.	Using the electrospun technique.	[59]
Inzana et al. in 2014.	Bone regeneration.	Calcium phosphate and collagen scaffolds	Using 3D printing technique to control the shape of scaffold.	[60]
Vikingsson et al. in 2015.	Articular cartilage regeneration.	Polycaprolactone-polyvinyl alcohol.	The composite scaffold possesses great potential for articular cartilage.	[61]
Na et al. in 2016.	Dental pulp regeneration.	3D stem cell sheet-derived pellet.	Odontogenic stem cells used for designing 3D stem cell sheet-derived pellet.	[62]
Lastra et al. in 2018.	Osteochondrogenesis regeneration.	Copolymer chitosan crosslinked scaffold	The nanostructured scaffold was highly biocompatible and non-cytotoxic.	[63]
Ghosh et al. in 2019.	For bone repair and regeneration.	Injectable alginate–peptide scaffolds	The scaffold served as a biomaterial for bone regeneration.	[64]
ElSheshtawy et al. in 2020.	Endodontics regeneration.	Plateletrich plasma-based scaffold	Using 2D radiographs and cone-beam computed tomography.	[65]
Zeng et al. in 2021.	Retinal cell culture.	Polycaprolactone scaffolds.	Biomimetic kerateine aerogel electrospun scaffolds.	[66]

**Table 2:** Biocompatibility and cytotoxicity of biopolymers in tissue engineering applications.

Biopolymeric Scaffold	Cell Type	Conclusion	Ref
3D porous cellulose scaffolds.	Osteoblast-like MG-63 cells.	The scaffold did not show any cytotoxic effect.	[87]
Non-covalent sericin–chitosan scaffold.	Human dermal fibroblasts.	No cytotoxic effect for the scaffold was observed against the human skin cells.	[88]
Recombinant collagen/hyaluronic acid composite scaffolds.	Mouse fibroblasts cells (L929 cells).	No cytotoxicity and good biodegradability was observed.	[89]
Collagen- and elastin-based scaffolds.	Human umbilical vein endothelial cells.	The scaffolds were highly compatible and non-cytotoxic.	[90]
Silk fibroin-based scaffolds.	Human fibroblast cells (GM07492).	High cellular viability and seemed to be non-cytotoxic.	[91]
Propolis/sodium alginate scaffolds.	Human dermal fibroblasts (HFFF2).	The scaffolds were non-toxic at low concentrations.	[92]
Gelatin hydrogels tissue scaffold.	Human pre-adipocytes (3T3-L1).	The scaffolds showed no cytotoxic effects on the cells.	[93]
Nanocellulose- and elastin-based scaffolds.	Human fibroblast cells.	All the prepared scaffolds seemed to be non-cytotoxic and biocompatible.	[94]
Hyaluronic acid/corn silk extract scaffold.	Mesenchymal stem cells.	High cellular differentiation without any cytotoxic effect.	[95]
Salt leached silk fibroin-based scaffolds.	Human adipose stem cells.	The scaffolds were highly biocompatible and non-cytotoxic.	[96]

**Table 3:** Summary of the most used techniques for biopolymer aerogel scaffold fabrication.

Technique	Principal	Ref
Electrospinning technique	Charged threads of biopolymeric solution or biopolymer melt are drawn using a special machine by high voltage electricity.	[114]
Solvent casting and practical leaching technique	Dissolving the polymeric powder in suitable solvents containing salt particles, which are then evaporated with the salts leaching out.	[115]
Freeze-drying technique	Freezing the dissolved polymer hydrogel and drying it under the vacuum to maintain the structural integrity of the hydrogel.	[116]
Stereolithography technique	Computer-aided technique prints photosensitive liquid of biopolymer layer-by-layer using an ultraviolet laser.	[117]
Injection molding technique	Melting and injecting the biopolymeric material into a mold, after which it cools and solidifies.	[118]
Gas foaming technique	Dissolving the biopolymer in organic solvents and then inserting gases used to pressurize the modelled until it is full of gas bubbles.	[119]
Selective laser sintering technique	The biopolymeric solution is printed by selective laser, which sinters the material in thin layers leading to 3D scaffold printing.	[120]
Fused deposition modelling technique	Deposition of biopolymeric materials extruded layer-by-layer through a special nozzle to form 3D multiple layers scaffolds.	[121]

## Data Availability

Data sharing not applicable. No new data were created or analyzed in this study. Data sharing is not applicable to this article.
